# The murine lung as a factory to produce secreted intrapulmonary and circulatory proteins

**DOI:** 10.1038/s41434-018-0025-8

**Published:** 2018-07-18

**Authors:** Michael C. Paul-Smith, Kamila M. Pytel, Jean-François Gelinas, Jenny McIntosh, Ian Pringle, Lee Davies, Mario Chan, Cuixiang Meng, Robyn Bell, Lidia Cammack, Caroline Moran, Loren Cameron, Makoto Inoue, Shu Tsugumine, Takashi Hironaka, Deborah R. Gill, Stephen C. Hyde, Amit Nathwani, Eric W. F. W. Alton, Uta Griesenbach

**Affiliations:** 1 Department of Gene Therapy, National Heart and Lung Institute, Imperial College London, London, UK; 2 UK Cystic Fibrosis Gene Therapy Consortium, London, UK; 3 Gene Medicine Research Group, Nuffield Division of Clinical Laboratory Sciences, Radcliffe Department of Medicine, University of Oxford, Oxford, UK; 4 Department of Haematology, University College London Cancer Institute, London, UK; 5 ID Pharma Co. Ltd. (DNAVEC Centre), Tsukuba, Japan

## Abstract

We have shown that a lentiviral vector (rSIV.F/HN) pseudotyped with the F and HN proteins from Sendai virus generates high levels of intracellular proteins after lung transduction. Here, we evaluate the use of rSIV.F/HN for production of secreted proteins. We assessed whether rSIV.F/HN transduction of the lung generates therapeutically relevant levels of secreted proteins in the lung and systemic circulation using human α1-anti-trypsin (hAAT) and factor VIII (hFVIII) as exemplars. Sedated mice were transduced with rSIV.F/HN carrying either the secreted reporter gene Gaussia luciferase or the hAAT or hFVIII cDNAs by nasal sniffing. rSIV.F/HN-hAAT transduction lead to therapeutically relevant hAAT levels (70 μg/ml) in epithelial lining fluid, with stable expression persisting for at least 19 months from a single application. Secreted proteins produced in the lung were released into the circulation and stable expression was detectable in blood. The levels of hFVIII in murine blood approached therapeutically relevant targets. rSIV.F/HN was also able to produce secreted hAAT and hFVIII in transduced human primary airway cells. rSIV.F/HN transduction of the murine lungs leads to long-lasting and therapeutically relevant levels of secreted proteins in the lung and systemic circulation. These data broaden the use of this vector platform for a large range of disease indications.

## Introduction

Our ongoing efforts to improve pulmonary gene transfer for the treatment of lung diseases such as cystic fibrosis (CF), have led to the development of a novel lentiviral vector (simian immunodeficiency virus [SIV]) pseudotyped with the Sendai virus (SeV) envelope proteins F and HN (recombinant (r)SIV.F/HN). This vector can drive high, stable levels of reporter gene expression in the lungs and nose of mice for the duration of their lifetime (~ 2 yr) and, in contrast to other viral vectors, repeated administration does not lead to loss of efficacy [1–3]. Similar expression profiles using lentivirus-mediated pulmonary gene transfer have been reported by others and are not unique to the F/HN pseudotype [4–6]. The efficacy and toxicity profile of the vector, therefore, supports progression toward the clinic.

We and others have previously shown that the nose and lung can also be used as efficient 'factory' for systemic protein production when transduced with a SeV vector or an adeno-associated viral vector [7, 8]. However, the transient gene expression and the lack of efficacy on repeated administration limit the use of SeV for translation into the clinic. The expression profile of rSIV.F/HN warranted a reappraisal of this strategy. Here, we explore the use of this platform technology, to secrete proteins either into the lungs or systemic circulation, using α1 antitrypsin (hAAT) and Factor VIII as exemplars, respectively.

hAAT is principally produced in the liver and released into the circulation. The protein has a wide range of functions but its primary role in the lung is the inhibition of neutrophil elastase (NE) [9]. Intravenous protein replacement therapy to treat α1 antitrypsin deficiency (AATD) lung disease is licensed in some countries, but there is debate over its efficacy [10–13]. Serum hAAT levels and disease severity vary dependent on the type of mutation [14] and AATD has been defined as a serum AAT below 11 µM, which translates into projected concentration in the airways of 1.1 µM (or 70 μg/l) [15]. These values provide 'therapeutic thresholds' for benchmarking new therapies. Importantly, patients with a SZ phenotype where AAT concentrations in epithelial lining fluid (ELF) are ~ 70 μg/ml do not develop lung disease, further supporting that this level of AAT is protective [15]. hAAT gene therapy clinical trials have largely focussed on intramuscular injection of adeno-associated virus (AAV), which has to date not produced these levels [16]. Production of hAAT directly in the lung may be a more efficient strategy to achieve therapeutic levels in the target organ, owing to the close proximity of lung epithelial cells and systemic circulation.

Haemophilia B patients have been successfully treated by intravenous administration of AAV carrying the factor IX (FIX) complementary DNA (cDNA) [17]. Replacement of the FVIII gene is more difficult to achieve because the standard cDNA is too large to fit into an AAV vector [18], although recent modifications have generated a shortened AAV-hFVIII construct [19] currently being assessed in clinical trials. Similar to AATD the 'therapeutic threshold' levels are clearly defined, with levels above 1% of normal reducing the frequency of bleeding episodes [18]. Here, we assess the capability of the rSIV.F/HN platform technology to achieve long-lasting therapeutic levels of these two exemplar proteins.

## Results

### Non-viral gene transfer does not reach therapeutically relevant levels of hAAT

We have previously shown that the cationic lipid formulation GL67A not only stabilises lung disease in CF patients [20], but also leads to expression of the secreted reporter gene Gaussia luciferase (GLux) in murine and ovine lungs [21]. Here, we assessed whether GL67A-mediated transfection also leads to significant levels of hAAT expression in murine lung.

**Fig. 1:**
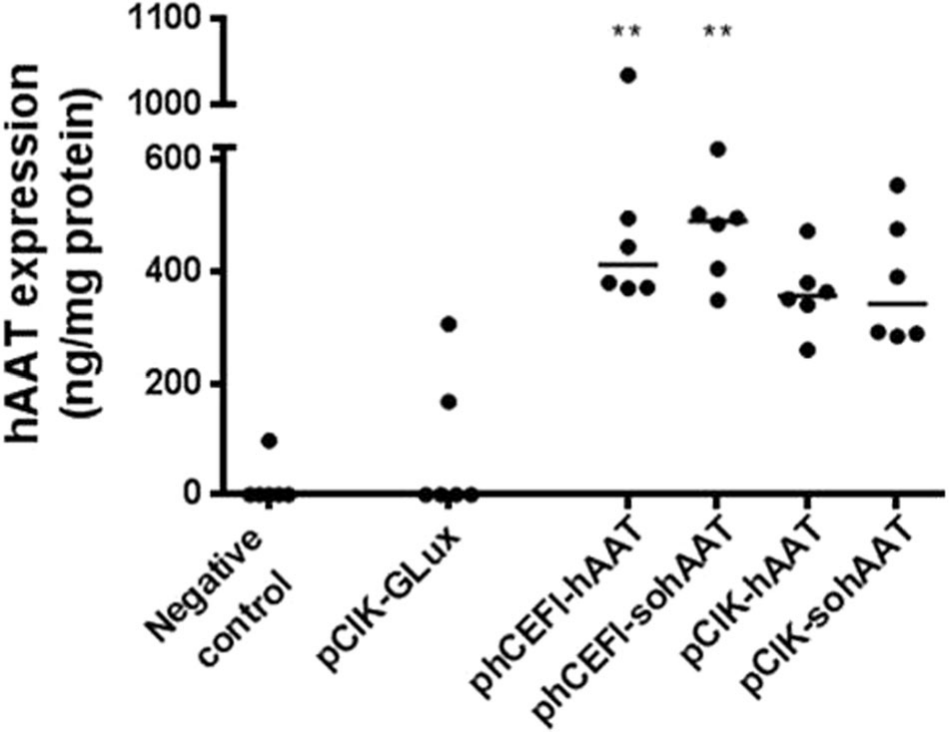
Production of human α_1_-antitrypsin following non-viral mediated gene transfer in vitro. Human embryonic kidney (HEK) 293T cells were transfected with plasmids expressing human α_1_-antitrypsin. Plasmids expressing either hAAT or sohAAT cDNAs (pCIK-hAAT, pCIK-sohAAT, phCEFI-hAAT, and phCEFI-sohAAT; Table 1) were complexed with LF2000. Plasmid pCIK-GLux was included as a negative control. Human AAT expression in cell culture supernatant was quantified 48 h post transduction and normalised to total protein content in each well. Each data point represents one well. The horizontal bars represent group medians. ** = *p* < 0.01 compared with control (Kruskal–Wallis test followed by Dunn multiple comparison post hoc test).The remaining groups were not significant following correction for multiple comparison

Plasmids that are depleted of CpGs (CG dinucleotides) reduce inflammation and achieve longer-lasting gene expression compared with CpG-containing plasmids in mouse lung in vivo [22] and we therefore removed all CpGs from the hAAT cDNA-generating sohAAT. We first confirmed that CpG-depletion did not negatively affect protein production in vitro. CpG-containing hAAT and CpG-free sohAAT cDNAs were cloned into plasmids carrying either a CMV promoter/enhancer (pCIK-hAAT, pCIK-sohAAT) or the CpG-free hCEFI regulatory element [22], which consists of the human CMV enhancer coupled to the elongation factor 1α promoter, (phCEFI-hAAT phCEFI-sohAAT). As shown in Fig. 1 all plasmids produced similar levels of hAAT after GL67A transfection into HEK293T cells in vitro.

**Fig. 2:**
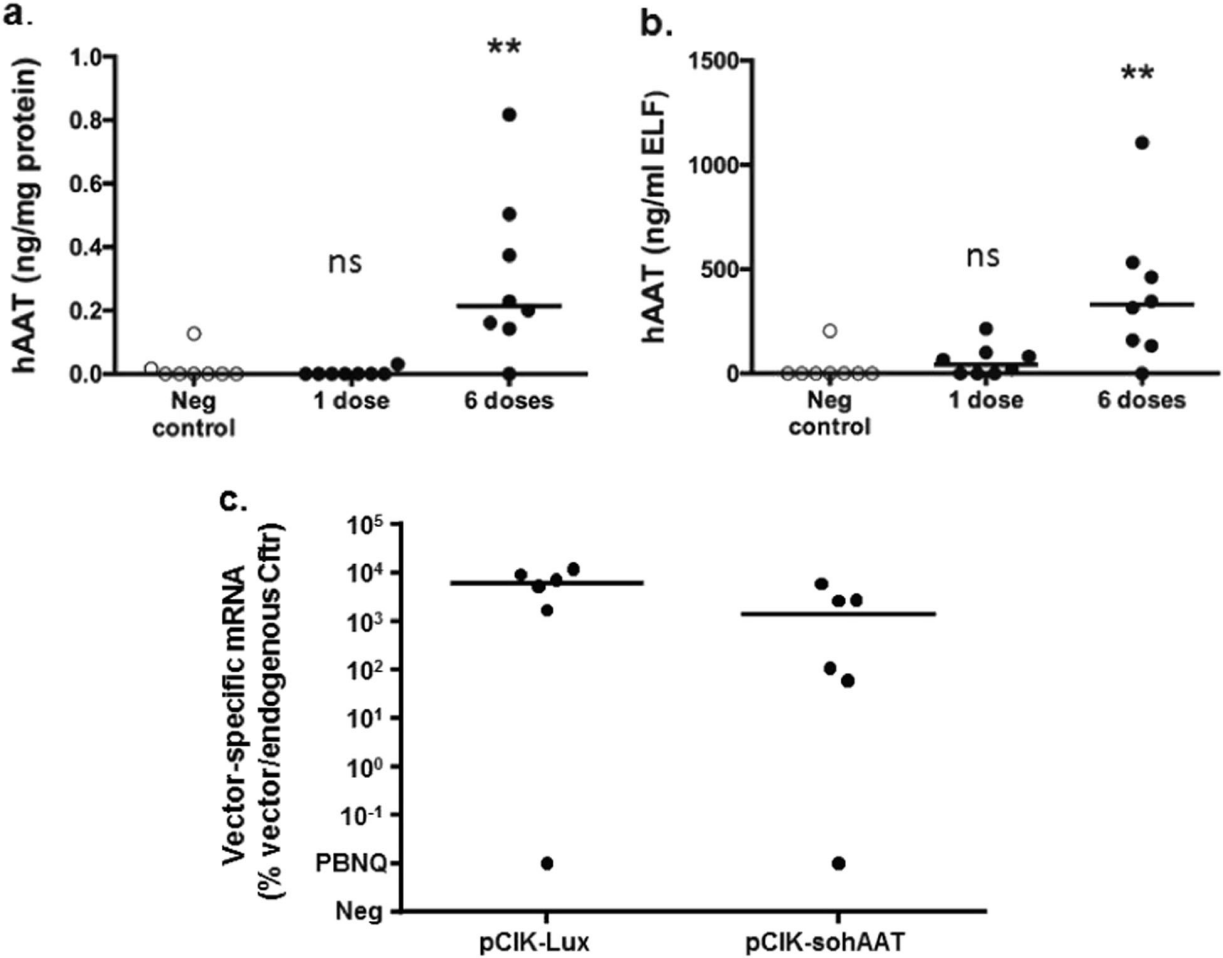
Significant but sub-therapeutic expression of human α_1_-antitrypsin following non-viral gene transfer to the lung. BALB/c mice were treated with one or six doses of GL67:phCEFI-sohAAT (80 μg/dose/mouse) or negative control (PBS) by nasal sniffing. Animals were harvested 6 days after the final dose and human α_1_-antitrypsin (hAAT) expression quantified in **a** lung tissue homogenate and **b** epithelial lining fluid (ELF). Each data point represents one animal; horizontal bars represent group medias. ** = *p* < 0.01. ns = not significant (Kruskal–Wallis test followed by Dunn multiple comparison post hoc test). **c** In a separate experiment BALB/c mice were treated with GL67A complexed with plasmids carrying *Firefly* luciferase (Lux; pCIK-Lux) or sohAAT (pCIK-sohAAT) cDNAs. Lungs were harvested 48 h post transduction, and qRT-PCR performed on total RNA. Data are expressed as percentage vector mRNA compared with endogenous mRNA (murine cystic fibrosis transmembrane conductance regulator, mCftr). Each data point represents one animal. Horizontal bars represent group medians. PBNQ = positive but not quantifiable, ns = not significant

CpG-free plasmid phCEF-sohAAT, expressing hAAT under the control of hCEFI, complexed with the cationic lipid GL67A was selected for subsequent in vivo experiments to assess whether non-viral gene transfer can reach therapeutically relevant levels of hAAT in murine lungs. Mice received either a single dose or six doses of phCEFI-sohAAT/GL67A (80 μg DNA in 100 μl per dose) at 7–10 day intervals by nasal sniffing. Control mice were treated with Dulbecco's phosphate-buffered saline (D-PBS). hAAT expression measured in lung homogenate (Fig. 2a) and ELF (Fig. 2b) 7 days after the final dose and showed that although dose-related hAAT expression was detectable in lung and ELF, the median concentration of hAAT in ELF was ~ 200-fold below the therapeutic target of 70 µg/ml.

To exclude the possibility that low-level hAAT expression in mice was owing to poor transfection efficiency, mice were treated with plasmid DNA carrying the soAAT (pCIK-sohAAT) or a Firefly luciferase (Lux) cDNA (pCIK-Lux) (both under the control of a CMV promoter/enhancer) complexed with GL67A (80 μg DNA in 100 μl). hAAT and Lux protein expression was quantified, as well as vector-specific mRNA by qRT-PCR. Mice treated with pCIK-Lux had > 4 log orders higher Lux expression than controls (*pCIK-lux*: median 33829 (range: 1160–42228) relative light units (RLU)/mg protein, negative control: median 1 (range: 1–2.6) RLU/mg protein, *n* = 6/group, *p* < 0.05), which was in line with our previous data [21], whereas hAAT expression in pCIK-sohAAT-treated mice was not different from controls (data not shown). Importantly, both pCIK-sohAAT and pCIK-Lux-treated mice expressed similar levels of vector-specific mRNA (Fig. 2c), clearly indicating that transfection efficiency was similar in both groups.

These experiments also indicated that luminescence-based luciferase assays, which detect robust levels of gene expression even after only a single dose of non-viral gene transfer [23], are log orders more sensitive than the hAAT enzyme-linked immunosorbent assay (ELISA) used for detection of protein expression after in vivo gene transfer.

### rSIV.F/HN generates high-level and persistent expression of secreted reporter genes in the murine lung

We next assessed whether rSIV.F/HN-mediated airway gene transfer leads to production of secreted proteins in vivo. Given the higher sensitivity of luminescence-based assays noted above, we first used a lentivirus carrying the secreted *GLux* gene, which we have previously shown to be a sensitive reporter protein in the context of GL67A-mediated airway gene transfer [24].

**Fig. 3:**
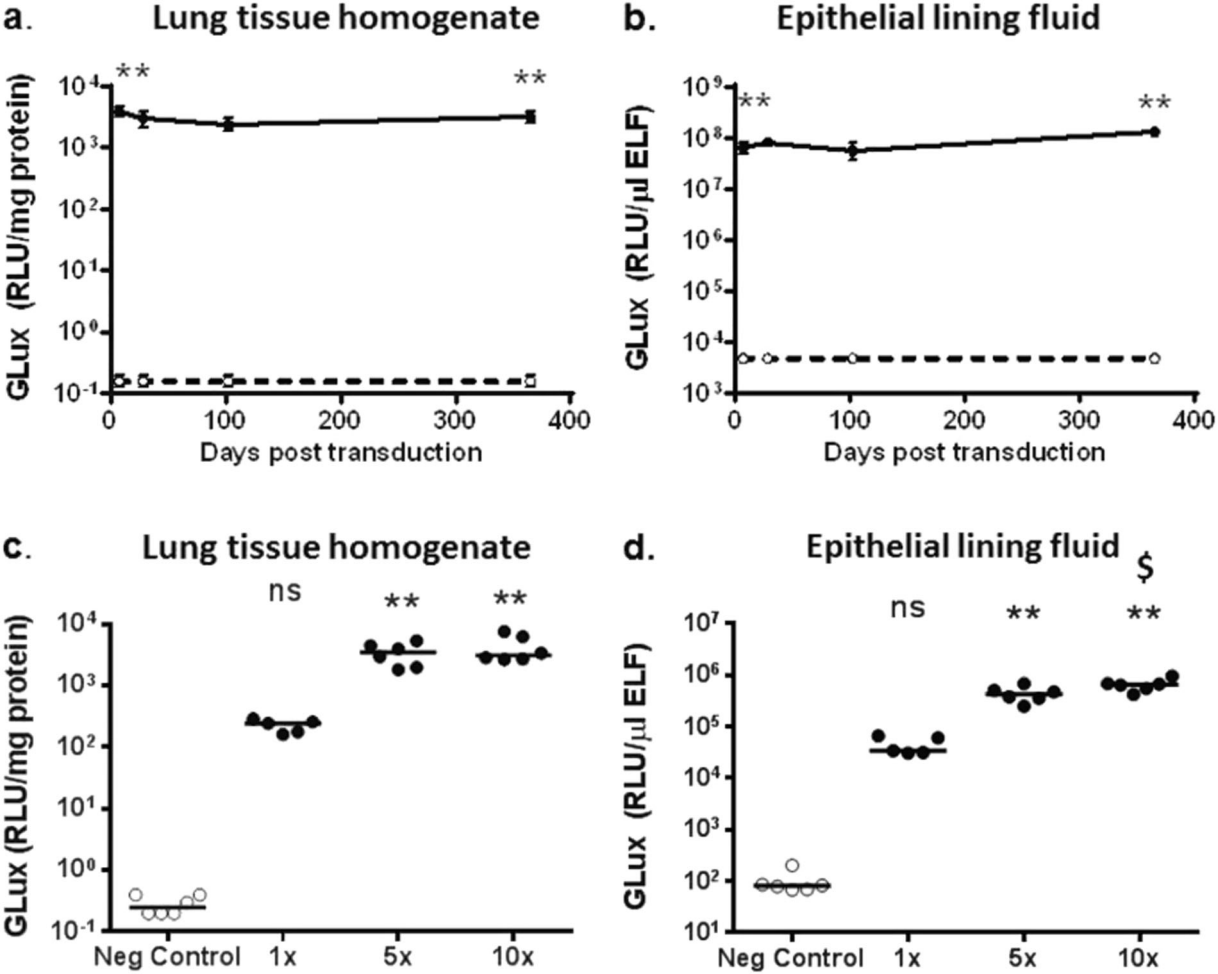
Sustained and dose-related expression of Gaussia luciferase (GLux) in mice after lentivirus transduction. C57Bl/6 mice were transduced with rSIV.F/HN carrying the soGLux cDNA under the control of the hCEF promoter (rSIV.F/HN-hCEF-soGLux, 1e7 TU/mouse, solid line) or received D-PBS (negative control, dotted line) by nasal sniffing and were culled between 7 and 365 days post transduction. GLux expression was measured in (**a**) lung tissue homogenate and **b** epithelial lining fluid (ELF). Data are expressed as mean ± SEM, *n* = 5–6 per group. ** = *p* < 0.01 compared with the negative control (only the early and late time-point were compared statistically using analysis of variance followed by a Bonferroni post hoc test. In a separate experiment using a different batch of virus mice were transduced with one, five or 10 doses of rSIV.F/HN-hCEF-soGLux and culled 7 days after the last dose. Owing to technical reasons the titre of the batch of virus could not be determined, but this is unlikely to affect the interpretation of the data. Negative control animals were treated with 10 doses of D-PBS. GLux expression was measured in (**c**) lung tissue homogenate and **d** ELF. Each dot represents one animal and the horizontal line represents the group mean. Data are expressed as mean ± SEM, *n* = 5–6 per group. RLU = relative light units. ** = *p* < 0.01 compared with negative control. $ = *p* < 0.05 compared with 5 × ns = not significant following correction for multiple comparison using analysis of variance followed by a Bonferroni post hoc test

Mice were transduced with rSIV.F/HN-hCEF-soGLux (1e7 TU/mouse) or received D-PBS, and tissues were harvested 7, 28, 102 and 365 days post gene transfer (*n* = 5–6/time-point/group). Seven days after transduction GLux expression was ~ 4 log orders higher (*p* < 0.01) in lung tissue (Fig. 3a) and ELF (Fig. 3b) compared with control mice. Importantly, expression was stable for at least 12 months in both lung tissue and ELF.

### Dose-related GLux expression after repeated administration of rSIV.F/HN

To address whether expression levels of a secreted protein increase in lung and ELF with repeated doses over a short period, mice were treated with 1, 5 or 10 doses of rSIV.F/HN-hCEF-soGLux over a 12-day period and culled 7 days after the final dose. Control animals were treated with 10 doses of D-PBS. All transduced mice produced GLux in lung tissue and ELF. There was a dose-related (*p* < 0.01) increase in GLux expression in lung tissue and in ELF (Fig. 3c, d). Even a modest doubling of the dose from 5 to 10 administrations did significantly (*p* < 0.05) increase expression levels in ELF.

### rSIV.F/HN-mediated transduction generates therapeutically relevant levels of hAAT in the lung

**Fig. 4:**
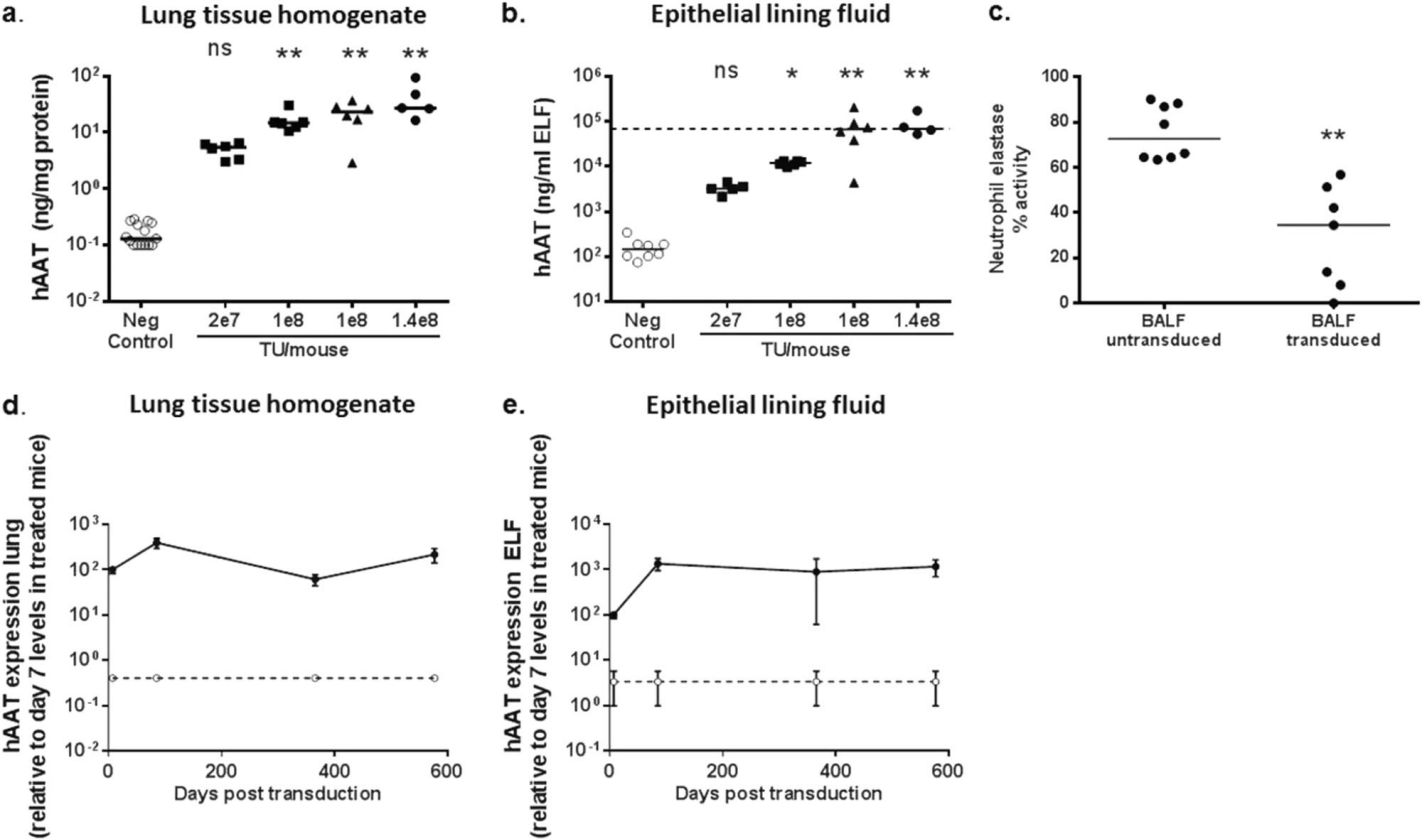
Sustained expression of α1 antitrypsin (hAAT) after lentivirus transduction. C57Bl/6 mice were transduced with rSIV.F/HN-hCEF-sohAAT (2e7–1.4e8 TU/mouse) or D-PBS (negative control) by nasal sniffing and culled ~ 10 days post transduction (*n* = 5–6/group). Different batches of virus were used for this experiment. Squares = virus grown in adherent culture (batch 1). Triangles = virus grown in suspension culture (batch 2). Circles = virus grown in adherent culture (batch 3). Human AAT expression was quantified in lung tissue homogenate (**a**) and epithelial lining fluid (ELF) (**b**). Each data point represents an individual animal and horizontal bars indicate the group median. The horizontal dashed line represents the therapeutic target level. Data are collated from three independent experiments performed on separate days. * = *p* < 0.05 and ** = *p* < 0.01 compared with negative control, respectively, using analysis of variance followed by a Bonferroni post hoc test. ns = not significant. Two cohorts of mice were treated with 1e8 TU/mouse because two separate batches of virus produced in either adherent or suspension culture were used (see Material and Methods). **c** A neutrophil elastase activity assay was performed on bronchoalveolar lavage fluid (BALF) from randomly selected mice (from Fig. 2a, b) treated with rSIV.F/HN-hCEF-sohAAT or D-PBS treated control animals. Each data point represents an individual animal, horizontal bars represent the group median. Data are expressed as % NE activity with the 'no sample assay control' set at 100%. ** = *p* < 0.01 compared with BALF from transduced control animals. (Kruskal–Wallis test followed by Dunn multiple comparison post hoc test). In a separate experiment, mice (*n* = 4–8/group) were transduced with rSIV.F/HN-hCEF-sohAAT (2e7 TU/mouse, solid line) or D-PBS (negative control, dotted line) by nasal sniffing and culled 7 days, 3, 12 and 19 months post transduction. Human AAT expression was quantified in lung tissue homogenate **d** and ELF **e** and expressed relative to day 7 levels of treated mice as a percentage of the day 7 time-point (absolute levels of AAT expression were similar to levels shown in Fig. 4a, b). Data are shown as mean ± SEM. ** = *p* < 0.01 compared with negative control at all time-points using analysis of variance followed by a Bonferroni post hoc test

We next assessed hAAT production after transduction of mouse lung with a single dose of rSIV.F/HN-hCEF-sohAAT (2e7–1.4e8 TU/mouse) or D-PBS; mice were culled 7–10 days post transduction (three independent experiments combined). hAAT expression was dose-related in both lung tissue (Fig. 4a) and ELF (Fig. 4b, Spearman rank *r* = 0.7, *p* < 0.01 for lung and ELF). Importantly, hAAT expression reached the therapeutic target of ~ 70 μg/ml in ELF of mice treated with ≥ 1e8 TU/mouse.

To determine whether the recombinant hAAT was functional we performed a NE inhibition assay using bronchoalveolar lavage fluid (BALF) of mice treated with rSIV.F/HN-hCEF-sohAAT (~ 1e8 TU/mouse, *n* = 7). NE activity was significantly (*p* < 0.01) inhibited in BALF of transduced mice when compared with D-PBS-treated controls (Fig. 4c). However, this in vitro assay does not allow us to compare the magnitude of inhibition to an in vivo environment.

### rSIV.F/HN-mediated transduction generates long-lasting hAAT expression in lung and ELF

To determine the duration of hAAT expression after a single dose, mice were treated with rSIV.F/HN-hCEF-sohAAT (2e7 TU/mouse) and culled ~ 7 days, 3, 12 and 19 months post transduction. hAAT expression was > 2 log orders higher compared with controls throughout the duration of the study and remained stable over the study period of ~ 19 months (Fig. 4d, e). A single dose of rSIV.F/HN-hCEF-sohAAT, therefore, leads to stable hAAT expression in lung and ELF for the lifetime of the animal.

hAAT expression levels over time appear more variable than Glux levels, despite the fact that the same promoter/enhancer (hCEF) was used in both constructs, reasons for this are currently unknown.

### Expression of proteins in serum following SIV-mediated gene transfer to the lung

**Fig. 5:**
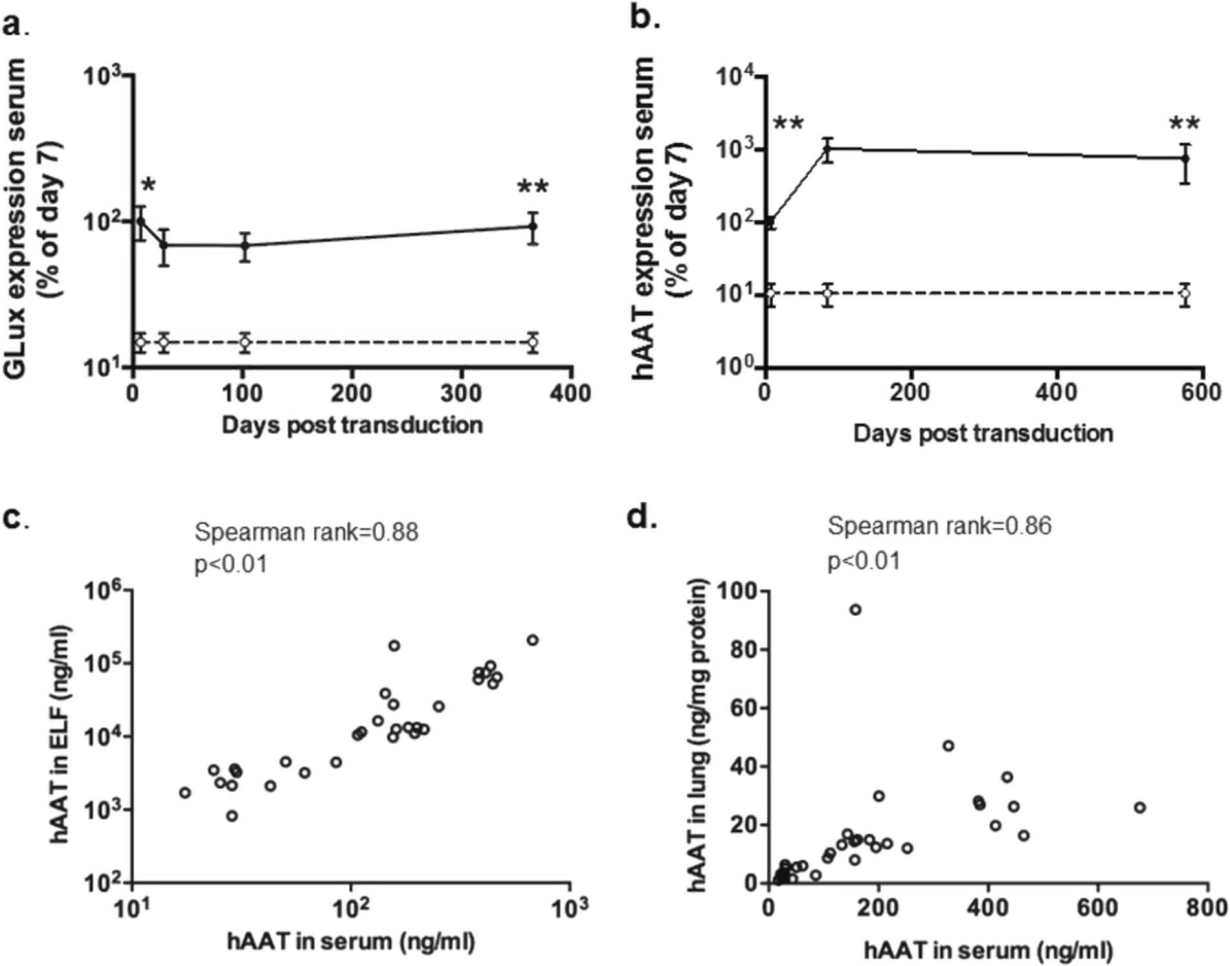
Release of recombinant proteins from lung into the circulation. **a** C57Bl/6 mice were transduced with rSIV.F/HN-expressing Gaussia luciferase (rSIV.F/HN-hCEF-soGLux, 1e7 TU/mouse, solid line) or D-PBS (negative control, dotted line) by nasal sniffing and culled between 7 and 365 days post transduction. GLux was quantified in serum and expressed as a percentage of day 7 values. Data are shown as mean ± SEM., *n* = 5–6/group/time-point. RLU = relative light units. * = *p* < 0.05, ** = *p* < 0.01 compared with negative control, respectively (only the early and late time-point were compare statistically), **b** C57Bl/6 mice were transduced with rSIV.F/HN-expressing hAAT (rSIV.F/HN-hCEF-sohAAT, 2e7 TU/mouse, solid line) or D-PBS (negative control, dotted line) (*n* = 4–8/group/time-point) by nasal sniffing and culled at the indicated time-points post transduction. Human AAT expression was quantified in serum and expressed as a percentage of day 7 values. Data are shown as mean ± SEM. ** = *p* < 0.01 compared with controls (only the early and late time-point were compare statistically) using analysis of variance followed by a Bonferroni post hoc test. **c** Correlation between hAAT in serum and epithelial lining fluid (ELF) and **d** serum and lung tissue homogenate. Each data point represents one animal

We have previously shown that SeV-mediated gene transfer to the murine airways leads to release of the recombinant protein into the circulation [7]. Here, we show that rSIV.F/HN-mediated gene transfer also leads to significant (*p* < 0.01) release of GLux from the lung into the circulation (Fig. 5a). In contrast to the transient SeV-mediated gene expression in blood, which only persisted for ~ 1 week [7], rSIV.F/HN expression in the systemic circulation remained stable during the 12 month study period (Fig. 5a). This expression profile was largely reproducible when measuring hAAT in mice transduced with rSIV.F/HN-hCEF-sohAAT (Fig. 5b). hAAT levels in serum were highly correlated with levels in lung tissue and ELF (Lung tissue: Spearman rank *r* = 0.86, *p* < 0.01, ELF: Spearman rank *r* = 0.88, *p* < 0.01) (Fig. 5c, d). These data suggest that hAAT serum levels are predictive of levels in lung and ELF and imply that noninvasive monitoring of pulmonary hAAT expression may be feasible.

### Expression of human factor VIII in plasma following gene transfer to the lung

To build on the finding that rSIV.F/HN lung gene transfer leads to release of the recombinant protein into the circulation, we investigated whether rSIV.F/HN-mediated gene transfer can generate a therapeutically relevant circulating blood protein and used the human blood clotting Factor VIII (hFVIII) as an exemplar.

**Fig. 6:**
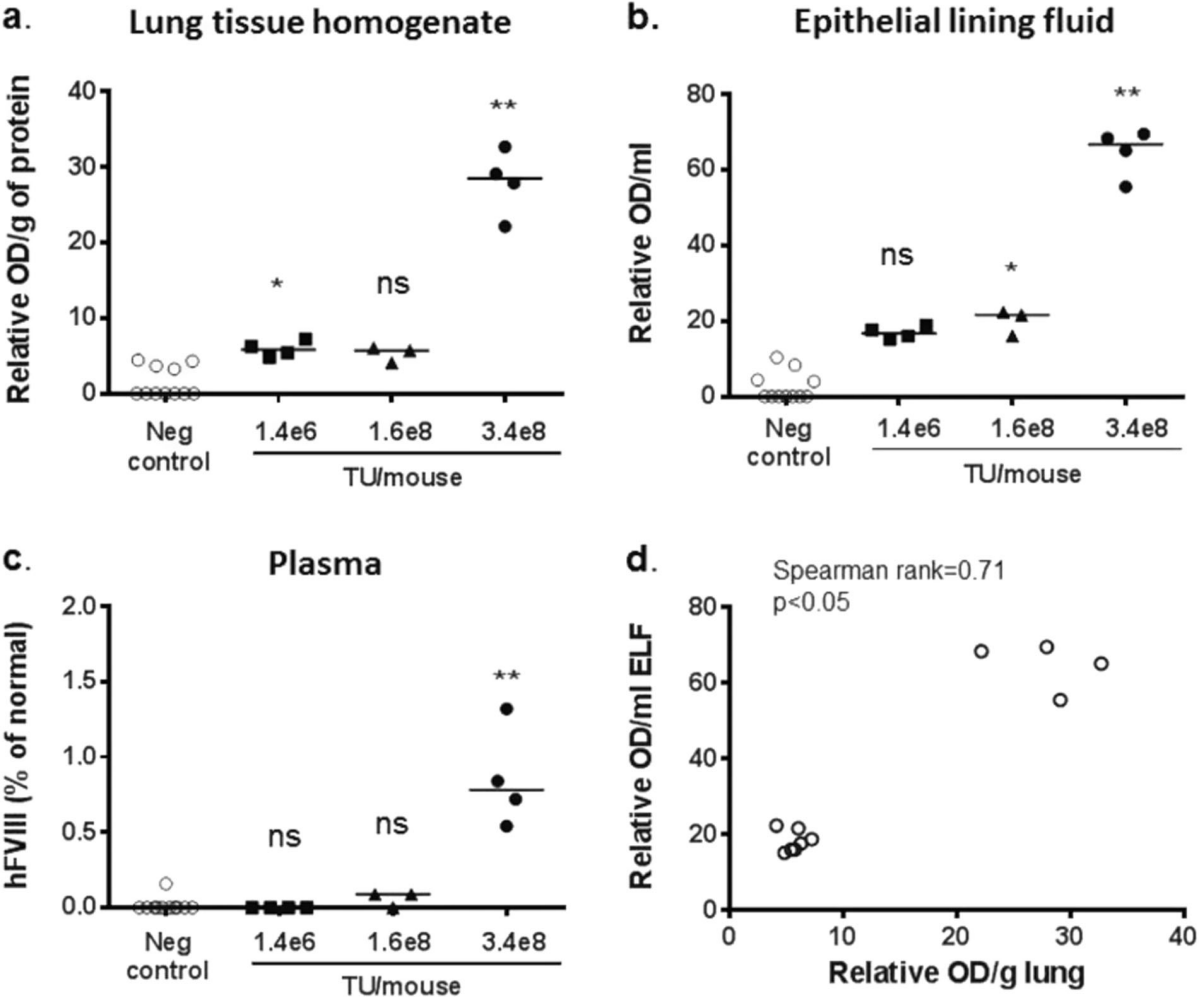
Expression of FVIII in lung, epithelial lining fluid and serum after lentivirus-mediated gene transfer in the lung. C57BL/6 mice were transduced with rSIV.F/H expressing human FVIII (rSIV.F/HN-CMV-FVIII-N6, 1.4e6 to 3.4e8 transduction units (TU)/mouse) or treated with D-PBS (negative control) by nasal sniffing (*n* = 3–4/group) and culled 10 days (1.4e6 and 1.6e8 TU/mouse) or 28 days (3.4e8 TU/mouse) after transduction. Different batches of virus were used for this experiment. Squares = virus grown in adherent culture (batch 1). Triangles = virus grown in adherent culture (batch 2). Circles = virus grown in suspension culture (batch 3). Human FVIII expression was quantified in lung tissue homogenate (**a**) epithelial lining fluid (ELF) (**b**) and plasma **c**. Each data point represents an individual animal. The horizontal bar represents the group median. * = *p* < 0.05, ** = *p* < 0.01 compared with negative control using analysis of variance followed by a Bonferroni post hoc test. ns = not significant following correction for multiple comparison. **d** Correlation between hFVIII in lung tissue and epithelial lining fluid (ELF). Each data point represents one animal

We first confirmed that a rSIV.F/HN virus carrying the human FVIII cDNA (hFVIII-N6) under the control of a CMV promoter/enhancer produced hFVIII in vitro (data not shown). Next, we transduced mice with rSIV.F/HN-CMV-hFVIII-N6 (1.4e6–3.4e8 TU/mouse) by nasal sniffing, collected lung tissue, BALF and blood between 10 and 28 days post transduction and compared hFVIII expression with untreated controls. Human FVIII expression in lung tissue and ELF was dose-related (lung: Spearman rank *r* = 0.67, *p* < 0.05, ELF: Spearman rank *r* = 0.86, *p* < 0.01) (Fig. 6a, b). However, hFVIII was only detectable in blood of mice treated with the highest available virus titre (Fig. 6c) approaching therapeutically relevant values (~ 1%). Again, there was a strong correlation between hFVIII in lung and ELF (Spearman rank *r* = 0.71, *p* < 0.05) (Fig. 6d) and, although *n* numbers were low, a significant correlation was also observed when comparing FVIII in blood and lung (*r* = 0.67, *p* < 0.05) and blood and ELF (*r* = 0.79, *p* < 0.01).

### Production of secreted proteins following lentiviral transduction of primary airway epithelial cells

To assess production of secreted proteins in human tissue, primary uncultured nasal epithelial cells were transduced with rSIV.F/HN expressing either hAAT or hFVIII. Owing to the comparatively short duration of the experiment (limited by cell viability) we first assessed if pseudo-transfection (the presence of hAAT and hFVIII protein in the virus preparation) would interfere with data interpretation. We compared hAAT and hFVIII levels in samples 'spiked' with rSIV.F/HN carrying hAAT or hFVIII (at levels equivalent to the dose used for transduction of nasal cells) to 'unspiked' controls and showed that there was no carry-over of hAAT (spiked: 55.9 ± 0.25 ng/ml, unspiked: 58.9 ± 2.5 ng/ml, *n* = 3–6/group), whereas a small, but significant amount of hFVIII was detectable in the virus preparation (spiked: 62.8 ± 2.9%, unspiked: 1.01 ± 0.59%, *p* < 0.05, *n* = 3–6/group).

**Fig. 7:**
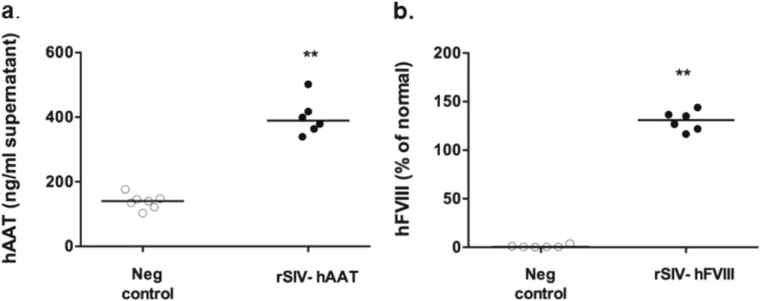
Production of secreted proteins in primary airway epithelial cells. Primary human nasal epithelial cells were transduced with rSIV.F/HN-expressing hAAT (rSIV.F/HN-hCEF-sohAAT, 1e6 transduction units (TU)/well) or hFVIII (rSIV.F/HN-CMV-FVIII-N6, 1e6 TU/well) or treated with D-PBS (negative control) (*n* = 6/group). Human AAT (**a**) and human FVIII (**b**) were quantified 24 h post transduction. Each symbol represents an individual sample. Horizontal bars indicate the group median. ** = *p* < 0.01 compared with negative control using analysis of variance followed by a Bonferroni post hoc test

Primary nasal epithelial cells transduced with rSIV.F/HN-expressing hAAT or hFVIII (*n* = 6/group) both expressed significant (*p* < 0.01) levels of therapeutic proteins (Fig. 7), indicating that rSIV.F/HN can generate therapeutically relevant secreted proteins in primary human airway epithelial cells.

## Discussion

Using the intracellular reporter gene Firefly luciferase, we have previously shown that pulmonary gene transfer using rSIV.F/HN leads to stable, persistent expression, is several log orders higher than the most efficient non-viral vector GL67A [2], and uniquely for a viral gene transfer agent retains efficacy when repeatedly administered. To broaden the potential use of this vector platform for a wide range of disease indications, we now show production of therapeutically relevant levels of exemplar secreted proteins in both the lung and circulation. In contrast to intracellular proteins such as CFTR, secreted proteins produced in the lung have the potential to exert benefits outside the target organ, which makes this strategy particularly attractive.

We have recently shown that repeated monthly application of GL67A-mediated expression of the CF protein CFTR can stabilise lung function in a double-blind, placebo-controlled trial [20]. Here, a single dose of rSIV.F/HN-expressing hAAT generated ~ 1000 times higher hAAT expression than six doses of GL67A. GL67A-mediated gene expression in ELF remained 200-fold below the therapeutic threshold of 1.1 µM (~70 µg/ml). The hCEF promoter/enhancer was used for both viral and non-viral gene transfer, as we had previously selected this promoter/enhancer for high-level GL67A and rSIV.F/HN gene transfer in the murine lungs [22, 25]. The vector transduces a range of cell types including ciliated airway epithelial cells, goblet cells, Club cells as well as alveolar type I and II pneumocytes [25], and may therefore generate therapeutically relevant levels of secreted proteins in different pulmonary and extra-pulmonary compartments.

We, and others have shown that, in contrast to adenovirus and AAV [26, 27], lentiviral vectors do not appear to lose efficacy on repeated administration (three monthly doses) [1, 2, 4] making the vector particularly attractive for the treatment of chronic diseases requiring life-long treatment. In contrast to monthly dosing, the daily administration regime assessed here, is unlikely to allow humoral and cellular immune responses to fully develop, but rather addresses a pragmatic issue for translation into clinical studies where volume restrictions for lung delivery may require doses to be split over several dosing days. We noted a dose-related increase in secreted protein expression in lung tissue and ELF, suggesting this strategy is feasible.

hAAT deficiency is an obvious disease target for AAT gene therapy. The first placebo-controlled trial assessing efficacy of inhaled hAAT has been completed, but results are not yet published in a peer reviewed journal. In the context of AAT gene therapy, clinical studies to date have focussed on intramuscular administration of an AAV vector-expressing hAAT [16]. Several clinical trials have been performed, but despite using high viral titres (6e12 vector genomes/kg) hAAT levels in blood have only reached ~ 3% of the therapeutic target [28, 29]. In an attempt to increase AAT expression in the lung, Halbert et al. [30] administered AAV6 topically to mice and dogs and demonstrated efficient, but transient gene expression due to an induced immune responses to the AAV capsid, which will also limit efficacy on re-administration of the vector. Chiuchiolo et al. [31] administered an AAV10 vector-expressing hAAT into the pleural cavity of mice and non-human primates, but did not report if therapeutic levels of AAT protein were achieved. Wilson et al. treated mice with a VSVG-pseudotyped lentivirus, which required co-delivery of the cationic lipid Lipofectamine 2000. The lipid/virus formulation transduced macrophages (but no other cell types) and led to persistent expression of AAT [32], but efficacy on repeated administration was not assessed.

In addition to AAT deficiency, overexpression of hAAT may also be of therapeutic value for a range of other neutrophil-driven lung inflammatory diseases such as cystic fibrosis and smoking-induced chronic obstructive pulmonary disease. rSIV.F/HN transduces a range of cell types including ciliated cells, goblet cells, Club cells and type I and II pneumocytes [25]. This broad cell type tropism may contribute to the efficient release of secreted proteins from the lungs into the circulation and may provide further opportunities for expanding hAAT-based treatments, which has recently been suggested for a range of diseases including type 2 diabetes, rheumatoid arthritis, and multiple sclerosis [33]. We have shown that the vector only rarely transduces progenitor basal cells [1, 25]. The persistent duration of expression is, however, compatible with the long half-life of terminally differentiated airway cells [34].

Long-term safety and efficacy of intravenous administration of an AAV8 vector-expressing factor IX in patients with haemophilia B has recently been demonstrated [17], but the development of a gene therapy-based treatment for haemophilia A has, in part, be hindered by the large size of the factor (F) VIII cDNA. In contrast to AAV, rSIV.F/HN can comfortably accommodate transgenes up to ~ 8 kb and Factor VIII was, therefore, chosen as an exemplar for producing a systemic protein in the lung. However, we envisage that this strategy may be easily adaptable for other diseases requiring expression of a systemic protein.

Prior to translation into clinical trials a number of points have to be addressed.

1. Gene transfer to the lung will require scale-up of good manufacturing process (GMP) rSIV.F/HN production methods. We have developed GMP compatible production methods based on transient transfection of serum-free suspension cultures (manuscript in preparation), which may readily be transferrable to a clinical manufacturing organisation.
2. We have shown here, and elsewhere, that rSIV.F/HN transduces fully differentiated human air liquid interface cultures, human lung slices and cells derived from human nasal brushings log orders more efficiently than GL67A [1, 2]. However, the question of efficacy and duration of gene expression in man remains to be determined.
3. We have previously shown that administration of rSIV.F/HN into the murine lung leads to mild neutrophilic inflammation [25]. The magnitude of this was similar to the inflammation induced by the non-viral formulation that was recently used in a Phase IIb multi-dose trial and which did not result in any significant toxicity in CF patients [20].
4. The risk of insertional mutagenesis owing to lentivirus integration into the genome of transduced cells is a potential concern and has been discussed in previous publications [2, 25]. To date, both clinical data from other lentiviral vector studies, as well as preclinical data generated by ourselves [25] and others support a good safety profile of these vectors.
5. We have previously shown that repeated administration of rSIV.F/HN to mouse lung is feasible despite detection of anti-rSIV.F/HN neutralising antibodies in serum [25]. It is currently unclear whether this is owing to (a) rapid cell entry thereby avoiding contact with neutralising antibodies, (b) serum-neutralising antibodies not being predictive of antibody levels in lung, (c) the in vitro neutralising antibody assay not being predictive of in vivo immunity or (d) other reasons.

In summary, these data suggest that rSIV.F/HN transduction of the lung may provide a new generic platform technology to produce long-lived, high levels of secreted proteins, relevant to both lung and systemic diseases. We envisage these proteins could include both those that are reduced or absent as a result of genetic or acquired defects, as well as therapeutic proteins currently administered as recombinant proteins.

## Material and methods

### Viral vector producer plasmids

rSIV.F/HN vectors were produced via transient transfection with a five plasmid split genome system derived from SIV African green monkey vervet isolate TYO-1 and SeV Z-strain (PMID: 12551999, PMID: 20332767). In brief, multiple vector genome plasmids encoding the packaged viral RNA were constructed containing a variety of transgenes and transcription elements. DNA fragments encoding FVIII-N6, and the codon-optimised, CpG-free cDNAs soGLux and sohAAT were inserted into unique NheI and ApaI restriction sites in the vector genome plasmid(1;3). The CMV [35] and hCEF [22] promoter/enhancer sequences, were incorporated into the vector genome plasmid via BglII and NheI restriction sites. The additional plasmids in the five plasmid system encoded SIV Gag/Pol, SIV Rev, SeV F protein derivative Fct4 and SeV HN protein derivative SIVct + HN [3]. All viruses used in this study are listed in Table 1.

**Table 1:** Plasmids and viral vectors used

Plasmid or virus name	Promoter/Enhancer	cDNA
phCEFI-hAAT	hCEFI	Alpha-1 antitrypsin (hAAT) cDNA
phCEFI-sohAAT	hCEFI	Codon-optimised CpG-depleted human alpha-1 antitrypsin (hAAT) cDNA
pCIK-hAAT	CMV	Alpha-1 antitrypsin (hAAT) cDNA
pCIK-sohAAT	CMV	Codon-optimised CpG-depleted human alpha-1 antitrypsin (hAAT) cDNA
pCIK-Lux	CMV	Firefly luciferase (Lux) cDNA
pCIK-GLux	CMV	Gaussia luciferase (Glux) cDNA
rSIV.F/HN-hCEF-soGlux	hCEF	Codon-optimised CpG-depleted GLux cDNA
rSIV.F/HN-hCEF-sohAAT	hCEF	Codon-optimised CpG-depleted hAAT cDNA
r.SIV.F/HN-CMV-FVIII-N6	CMV	codon-optimised FVIII containing 226 amino acid residues of the B domain

Note: for pragmatic reasons (availability), the CMV rather than the hCEF promoter/enhancer were used for all FVIII studies. The hCEF promoter is comprised of a CpG-free form of the human CMV immediately/early enhancer and a CpG-free form of the human Elongation Factor 1a promoter. Plasmids (phCEFI-hAAT and phCEFI-sohAAT also contain a synthetic recombinant intron

### Vector production and titration

Production of recombinant SIV vectors was performed using a five plasmid transient transfection method in adherent HEK293T cells as previously described [1]. In addition, one batch was generated using transient transfection of HEK293T cells grown in suspension and purified using a combination of anion exchange chromatography and tangential flow filtration [36]. The latter batch was used for the dose response experiment described in Fig. 4a, b. Squares = virus grown in adherent culture (batch 1). Triangles = virus grown in suspension culture (batch 2).

The viral particle titre (VP/ml) was determined essentially as described by Mitomo et al. [1], using Real-Time Quantitative PCR (Q-PCR) with primers spanning the WPRE sequence (Forward: TGGCGTGGTGTGCACTGT; Reverse: CCCGGAAAGGAGCTGACA; Probe: 6FAM-TTGCTGACGCAACCCCCACTGG-TAMRA). Virus RNA was prepared using QIAamp Viral RNA kit including carrier RNA (QIAGEN, Crawley, UK), followed by in-solution DNAse (Ambion, DNAfree) and quantified by one-step RT-q-PCR using QuantiTect (QIAGEN) against a standard curve of RNA mimics containing the WPRE sequence.

Functional titre, reported as transducing units per ml (TU/ml) was calculated following transduction of HEK293F cells with serial dilutions of viral vector and extraction of DNA using QIAamp blood DNA kit (QIAGEN). Viral DNA genomes were quantified by Q-PCR (same WPRE primers as above) against a standard curve of plasmid DNA containing the WPRE sequence, using TaqMan Universal Mastermix (Life Technologies), then normalised to total ng. DNA titre was calculated from the slope of the best-fit line on a plot of WPRE copies per well of cells against the volume of virus per well of cells.

### Plasmids and non-viral gene transfer agents

The hAAT cDNA was a gift from C. Halbert (Seattle, USA) and a codon-optimised, CpG-free version of the cDNA (sohAAT) was synthesised (GeneArt Regensburg, Germany). The human codon-optimised FVIII containing 226 amino acids of the B domain (FVIII-N6) has been described previously [19]. These cDNAs were inserted into the pCIK plasmid backbone (Promega, Southampton, UK) under the control of the CMVIE promoter, as well as the Firefly luciferase cDNA (Lux) and GLux cDNA [21, 35], or into a CpG-free plasmid backbone under the control of the CpG-free promoter hCEFI [22]. All plasmids are described in Table 1. Plasmids were produced using standard Qiagen kits (QIAGEN, Crawley, UK) according to the manufacturer’s recommendation. For in vitro and in vivo transfection experiments Lipofectamine 2000 (Life Technologies) and GL67A [37] were used, respectively (see below). Plasmid and liposome formulations were complexed for in vitro and in vivo gene transfer as previously described [23].

### In vitro transfection

Human embryonic kidney cells 293T/17 (ATCC, Manassas, VA, USA) were seeded in six-well plates (250,000 cells/well) in complete DMEM (Life Technologies, UK) containing 10% fetal calf serum (Sigma, Gillingham, UK) and 1% Pen/Strep (Sigma, UK). The cells were purchased ~ 3 years ago and have not been authenticated or mycoplasma tested since. After ~ 24 h cells in six-well plates were transfected with plasmids carrying the hAAT or sohAAT cDNA (1 µg pDNA/well in 400 µl total volume), or an irrelevant plasmid (pCIK-Glux as a negative control), complexed with LF2000 as previously described [21]. At 48 h post transduction hAAT was quantified in cell culture medium and expression corrected for total cell protein as described below.

### In vivo transfection and transduction

All animal procedures were performed in accordance with the conditions and limitations of the UK Home Office Project and Personal licence regulations under the Animal Scientific Procedure Act (1986). Female C57Bl/6 or Balb/C mice (6–8 weeks) purchased from Charles Rivers Laboratories, Tranent, UK, or Harlan, Bicester, UK) were used. Mice were randomly allocated to the different treatment groups. Analysis was performed in a non-blinded manner.

Mice were anaesthetised using isoflurane and treated with viral or non-viral vector (100 µl total volume/dose) by nasal sniffing as previously described [38]. In brief, mice are held upright and the mouth is closed by applying gentle pressure with the thumb. Liquid is applied to the nostrils and rapidly (few seconds) sniffed into the lung. Animals are placed on their front and regain consciousness within a couple of minutes. Figure legends indicate the virus titre used in each case, except for Fig. 1c, d where the virus titre was not available due to technical problems. Control animals received either D-PBS (100 µl) or an irrelevant rSIV.F/HN vector, as indicated in the figures. We have previously shown that negative control viruses do not lead to upregulation of endogenous murine AAT levels in mouse ELF (D-PBS treated: median 187.8 (range 114.8–342.8) pg hAAT/ml ELF, rSIV.F/HN-Lux: median 103.6 (range 74.8–342.8) pg hAAT/ml ELF). Treatment of negative controls with D-PBS led to an overall reduction of animals used in line with the 3Rs (replacement, reduction and refinement) guidelines, because the D-PBS-treated control animals could be used as controls for viral and non-viral gene transfer experiments.

For the non-viral gene transfer, mice received either one or six doses of phCEFI-sohAAT/GL67A or one dose of pCIK-Glux/GL67A (80 µg plasmid/dose, *n* = 8/group). Control mice were treated with D-PBS (*n* = 8/group). We have previously shown that negative control plasmids complexed with GL67A do not lead to upregulation of endogenous murine AAT levels in mouse ELF (D-PBS treated: median 75 pg (range 65.6–75.0) hAAT/ml ELF, pDNA/GL67A: median 130.4 pg (range 0–437) hAAT/ml ELF). As mentioned above, treatment of negative controls with D-PBS reduced the overall number of animals used. In some experiments non-viral gene expression was quantified by RT-q-PCR as previously described [39]. In brief, primers and probes for vector-specific mRNA were directed toward an intron in the 5’ untranslated region common to both plasmids used in the experiment (Forward: TGAGGCACTGGGCAGGTGT; Reverse: GTCGTATTAAGTACTCTAGCCTTAAGA; Probe: CCACTCCCAGTTCAATTACAGC); primers and probes for endogenous mRNA were directed to murine cystic fibrosis transmembrane conductance regulator (mCftr) (Forward: AGCCAGCTTTATCTCCAAACTCTTC; Reverse: GCTGTCTGTACCCTTTCCTCAAA; Probe: TCAGCTGGAGCACAGC). Negative control mice were matched for age, strain and sex.

At indicated time-points after the last dose, mice were culled and BALF, blood and lung tissue were collected and processed as previously described [21]. To control for dilution of ELF when performing bronchoalveolar lavage, a urea assay (Abcam, Cambridge, UK) was performed according to a standard method [40] on serum and BALF samples from 14 untreated mice. For each mouse we calculated by how much the ELF was diluted after lavage with PBS (dilution factor). The mean dilution factor in the 14 mice was calculated and used to calculate gene expression in ELF in treated animals.

### Transduction of primary human airway epithelial cells

Primary human airway epithelial cells were collected from healthy volunteers using a cytology brush as previously described [41]. Tissue collection was approved by the Royal Brompton and Harefield NHS Foundation Trust Research Ethics Committee and all subjects gave informed written consent. Cells were transduced and cultured as previously described [2]. At indicated time-points, cell culture medium was collected and hAAT or FVIII quantified as described below.

### Quantification of protein expression

#### GLux

GLux was quantified using a GLux assay kit (New England Biolabs, Herts, UK) as previously described [21]. For lung tissue homogenates, GLux expression was corrected for total cellular protein using the BioRad DC Protein Assay (BioRad, Hercules, CA, USA) as previously described [23] and data expressed as GLux RLU/mg total protein.

#### hAAT

A commercially available hAAT ELISA kit (Abcam; sensitivity 0.39–100 ng/ml) was used according to manufacturer’s recommendations. Expression of hAAT in lung tissue homogenates was corrected for total protein as described above.

#### hFVIII

A commercially available hFVIII ELISA kit (Diagnostica Stago, France; sensitivity 0.5–140% of normal hFVIII) was used according to manufacturer’s recommendations. hFVIII levels in blood are expressed as % of normal as recommended by the manufacturer, although this is not suitable for quantification of FVIII in lung and ELF. Expression of hFVIII in lung tissue homogenates was corrected for total cellular protein as described above and expressed as relative OD/g of total protein. Expression of hFVIII in ELF was expressed as relative OD/ml ELF. Expression of hFVIII in primary human airway epithelial cells was also expressed as % of normal.

For duration studies all data were expressed as a % ratio of day 7 levels, the first time-point that was measured.

### Neutrophil elastase inhibition assay

A NE colorimetric drug discovery kit (Enzo Life Sciences, Exeter, UK) was used to quantify NE activity according to the manufacturer’s recommendations. BALF from mice treated with rSIV.F/HN-hCEF-sohAAT (1e8–1.4e8 TTU/mouse) were analysed and compared with BALF from mice treated with D-PBS. Data are expressed as % NE activity with the 'no sample control' set at 100%.

### Statistical analysis

Sample sizes were determined based on previous experience with the vector. All samples were included into the analysis. Analysis of variance followed by a Bonferroni post hoc test, or Kruskal–Wallis test followed by Dunn multiple comparison post hoc test, was performed for multiple group comparison, after assessing parametric and nonparametric data distribution with the Kolmogorov–Smirnov normality test. An independent Student's *t* test or a Mann–Whitney test was performed for two-group parametric and nonparametric data as appropriate. Pearson correlation was performed for parametric data, and Spearman rank correlation performed for nonparametric data. All analyses were performed using GraphPad Prism 6 (GraphPad Software, Inc., La Jolla, CA USA) and the null hypothesis was rejected at *p* < 0.05.
